# Immediate effects of spinal manipulation on painful sensitivity and postural stability in patients with chronic nonspecific low back pain: study protocol for a controlled randomised clinical trial

**DOI:** 10.1186/s13063-022-06111-4

**Published:** 2022-03-03

**Authors:** João Paulo Freitas, Leticia Amaral Corrêa, Juliana Valentim Bittencourt, Karine Marcondes Armstrong, Leandro Alberto Calazans Nogueira

**Affiliations:** 1 Rehabilitation Science Postgraduation Program, Augusto Motta University Centre (UNISUAM), Rio de Janeiro, Brazil; 2 Physiotherapy Department, Midwestern Parana State University (UNICENTRO), Paraná, Brazil; 3 Physiotherapy Department, Guairacá University Centre (UNIGUAIRACA), Paraná, Brazil; 4 Physiotherapy Department, Federal Institute of Rio de Janeiro (IFRJ), Rio de Janeiro, Brazil

**Keywords:** Low back pain, Chronic pain, Postural balance, Musculoskeletal manipulations, Study protocol

## Abstract

**Background:**

Low back pain is one of the main public health concerns. Chronic low back pain (cLBP) reduces functional capacity and affects postural stability. Although health professionals widely use spinal manipulation, its immediate effect on painful sensitivity and postural stability is lacking. This study aims to verify the immediate effects of lumbar spinal manipulation on the pressure pain threshold and postural stability in individuals with cLBP.

**Methods:**

A two-arm, placebo-controlled clinical trial with parallel groups and examiner-blinded will be conducted with 80 participants with cLBP from an outpatient physical therapy department, randomly allocated at a 1:1 distribution. The experimental group will receive a lumbar spinal manipulation technique, and the placebo group will receive a simulated lumbar spinal manipulation. Both groups will receive one session of treatment and will be evaluated before and immediately after the intervention. The primary outcomes will be the pressure pain threshold and postural stability. Pain intensity and patient’s expectation will be assessed as a secondary outcome. The pressure pain threshold will be assessed using a pressure algometer in 6 different anatomical regions. The evaluation of postural stability will be performed in a baropodometry exam by displacing the centre of pressure. The pain intensity will be measured using the Numeric Pain Rating Scale. A Likert scale will be used for the patient’s expectation about the treatment. A two-way analysis of variance will compare the effect of the interventions between groups.

**Discussion:**

This study will provide insights regarding the immediate effects of spinal manipulation in patients with cLBP against a simulated spinal manipulation using objective outcomes and considering patients’ expectations regarding the treatment.

**Trial registration:**

Brazilian Registry of Clinical Trials RBR-3ksq2c. Registered on 13 July 2020

## Administrative information

Note: the numbers in curly brackets in this protocol refer to SPIRIT checklist item numbers. The order of the items has been modified to group similar items (see http://www.equator-network.org/reporting-guidelines/spirit-2013-statement-defining-standard-protocol-items-for-clinical-trials/).

**Table:** 

Title {1}	Immediate effects of spinal manipulation on painful sensitivity and postural stability in patients with chronic nonspecific low back pain: study protocol for a controlled randomised clinical trial.
Trial registration {2a and 2b}.	Brazilian Registry of Clinical Trials – RBR-3ksq2c WHO U1111-1252-3077
Protocol version {3}	Version 4 of 24-01-2022
Funding {4}	This research is funded in part by Coordenação de Aperfeiçoamento de Pessoal de Nível Superior – Brasil (CAPES) – Finance Code 001. Material support will be provided in part by Guairacá University Centre (UNIGUAIRACA)
Author details {5a}	J.P. Freitas: Rehabilitation Science Postgraduation Program – Augusto Motta University Centre (UNISUAM), Rio de Janeiro, Brazil. Physiotherapy Department – Midwestern Parana State University (UNICENTRO), Paraná, Brazil. Physiotherapy Department – Guairacá University Centre (UNIGUAIRACA), Paraná, Brazil. L.A. Corrêa: Rehabilitation Science Postgraduation Program – Augusto Motta University Centre (UNISUAM), Rio de Janeiro, Brazil. J.V. Bittencourt: Rehabilitation Science Postgraduation Program – Augusto Motta University Centre (UNISUAM), Rio de Janeiro, Brazil. K.M. Armstrong: Physiotherapy Department – Midwestern Parana State University (UNICENTRO), Paraná, Brazil. L.A.C Nogueira: Rehabilitation Science Postgraduation Program – Augusto Motta University Centre (UNISUAM), Rio de Janeiro, Brazil. Physiotherapy Department – Federal Institute of Rio de Janeiro (IFRJ), Rio de Janeiro, Brazil.
Name and contact information for the trial sponsor {5b}	Investigator initiated clinical trial; L.A.C Nogueira (Principal investigator) leandronogueira@souunisuam.com.br.
Role of sponsor {5c}	This is research initiated by the investigator. The funders played no role in the study design and in the collection, analysis, and interpretation of data and in the writing of the manuscript.

## Introduction

Low back pain (LBP) is a primary cause of disability despite the number of therapeutic options. LBP affects more than 500 million people globally [1], and 69% of them will experience a new episode of LBP in a year [2]. Although most acute LBP patients recover within a few weeks, about a quarter of patients who come to primary care develop chronic LBP (cLBP) [3]. Nonspecific cLBP is the most common type of LBP and has an unfavourable prognosis. People with nonspecific cLBP have moderate levels of pain and disability at 12 months [4]. Several approaches are available for the treatment of the cLBP, including spine manipulation. Spinal manipulative therapy has been recommended for LBP by many clinical practice guidelines [5–7]. However, definitive indications and mechanisms of spinal manipulative therapy are still not well established [8].

Spinal manipulative therapy is a passive manual technique used in the spine involving high velocity with low amplitude thrust applied to a joint complex within its range of motion [9]. Spinal manipulative therapy leads to short-term pain relief similar to other recommended therapies for cLBP [10, 11]. Likewise, patients with cLBP experience short-term improvement in function after a spinal manipulative therapy compared with non-recommended interventions or sham manipulation [10]. The effect of spinal manipulative therapy has contradictory findings compared to sham manipulation or placebo intervention [12]. Spinal manipulative therapy demonstrated a similar reduction in pain but superior improvement in function compared to sham manipulation [10]. On the other hand, spinal manipulative therapy had the identical effect of sham cold laser therapy for mild to moderate cLBP [13]. Thus, the type of placebo and the outcome measurement used may interfere in interpreting the effects of spinal manipulative therapy for cLBP.

Simulated procedures may lead to a placebo effect, which objective outcomes can evaluate. Simulated interventions are considered more appropriate as control [14]. Randomised clinical trials of spinal manipulative therapy using a simulated technique, maintaining the blindness of the participants, has already been shown to be possible [15]. Although placebo interventions are more effective than no intervention at short term in patients with LBP, objective outcome measures tend to be less influenced by the placebo effect than subjective measures [16]. An immediate or temporary improvement in LBP can be assessed objectively by the pressure pain threshold and postural stability. Spine manipulation affects the regional pressure pain threshold [17]. However, the pressure pain threshold did not alter immediately after spinal manipulation in patients with LBP in a previous study [18]. Postural stability appears impaired in individuals with nonspecific cLBP [19–23]. Thus, pressure pain threshold and postural stability may provide an objective measurement of the effects of spinal manipulative therapy over a simulated intervention.

Although spinal manipulative therapy has a small immediate effect on the functionality of patients with cLBP [24], there is no specific patient characteristic that identifies patients more likely to benefit from this intervention [25]. Recognising patients’ perceptions about the causes of pain and reducing the LBP is essentially better to guide clinical practice and future research [26, 27]. Patients with LBP consider temporary (hourly) relief an acceptable outcome, while clinical trials tend to consider an efficacy for long-term outcomes [27]. In addition, the short-term benefit may favour the performance of other measures with a higher level of evidence, such as exercising, since manual therapy seems to be better defined in association with physical exercise [7]. Therefore, we propose a randomised controlled clinical trial to (1) verify the immediate effects of lumbar spinal manipulation on the pressure pain threshold and postural stability in individuals with nonspecific cLBP. Secondarily, the study will (2) verify the immediate effect of lumbar spinal manipulation on the pain intensity in patients with nonspecific cLBP and to (3) verify if patient’s expectation about the treatment interferes with pressure pain threshold, postural stability, and pain intensity. We hypothesised that lumbar spinal manipulation would immediately increase the pressure pain threshold and improve postural stability in patients with cLBP. Besides, lumbar spinal manipulation would decrease pain intensity, and a positive patient’s expectation would affect pressure pain threshold, postural stability, and pain intensity.

## Methods

### Study design

A two-arm, placebo-controlled clinical trial with parallel groups and blinded examiner will be conducted following the checklist recommendations in Consolidated Standards of Reporting Trials (CONSORT) [28] and Standard Protocol Items: Recommendations for Interventional Trials (SPIRIT) [29].

### Registry

This trial was approved by the Research Ethics Committee of the State University of the Midwest – UNICENTRO (number 31299020.0.0000.0106), according to the guidelines of Resolution 466/12 of the National Health Council in accordance with the Helsinki Declaration for research in humans and registered in the Brazilian Registry of Clinical Trials (REBEC) (number RBR-3ksq2c).

### Setting

The trial will be conducted at the Guairacá Integrated Clinics, Guairacá University Centre (UNIGUAIRACA), in Guarapuava, PR, Brazil.

### Recruitment

Participants will be recruited by invitation established in an announcement in the Guairacá Integrated Clinics, as well as advertisements through social networks. Eligible patients with nonspecific cLBP will be invited to participate in the study. All subjects who agree to participate in the research must sign the informed consent form.

### Eligibility criteria

Participants with nonspecific cLBP (lasting at least 3 months); aged between 18 and 55 years, with moderate/severe current pain intensity (at least 3 points on the Numeric Pain Rating Scale); who are not undergoing physical therapy treatment for LBP; with no symptoms below the knee will be recruited to the study. The exclusion criteria adopted will be (1) widespread chronic pain; (2) ligament laxity or hyper flexibility; (3) pregnant women; (4) conditions that contraindicate the use of vertebral manipulation techniques at high speed and low amplitude (red flags) such as vertebral fractures, cauda equina syndrome, cancer, inflammatory rheumatic diseases, vertebral infections, and bone tuberculosis; (5) any condition that may interfere with pain sensitivity measures, for example, changes in skin sensitivity, neurological diseases, or psychiatric diseases; (6) any condition that interferes with body balance, for example, neurological diseases or vestibulopathy; and (7) score equal to or greater than 19 in the Brazilian version of PainDETECT questionnaire (Brazilian Portuguese Language) [30]. Participation in the trial will be voluntary, and the participants who refuse to participate or cannot complete the study for any reason will be considered drop-out.

### Recruitment and enrolment strategies

Participants will be recruited continuously until the desired sample size is reached. We will use different recruitment methods, including physical therapist referrals who assist the patients in outpatient physiotherapy, invitation established in an announcement in the Guairacá Integrated Clinics, as well as advertisements through social networks.

### Randomisation, allocation, blinding, and implementation procedures

Participants will be randomly allocated at a 1:1 distribution to one of two groups: the experimental group (group 1) will receive a lumbar spinal manipulation technique, and the placebo group (group 2) will receive a simulated lumbar spinal manipulation technique. The allocation sequence will be prepared a priori using the “Research Randomizer”, an online random number generator available at https://www.randomizer.org/. Participants will be allocated to the experimental group (group 1) or placebo group (group 2) using randomly permuted blocks of 4 and 6. Allocation will be concealed through sequentially numbered consecutively (1 to 80), sealed, opaque envelopes with an index card containing a sentence that will inform the examiner of the participant’s group allocation. An independent examiner not involved in the study recruitment, assessment, or data analysis will assign interventions to ensure secret allocation. The same examiner will open the sealed envelopes after the informed consent form has been completed, and the participant carries out the initial assessment. The participants will receive a unique study enrolment number and be referred to the physiotherapist responsible for the intervention that will perform spine manipulation or simulation of spine manipulation according to the group in which the participant was allocated.

After performing the pre-intervention evaluation, the examiner (examiner 1) will leave the evaluation room to remain blind to the intervention, and a physiotherapist (examiner 2) with experience in spinal manipulative therapy will enter the room to perform the manipulation technique or the simulated technique according to the previous randomisation. Examiner 2 will also be blinded to the outcome assessment. After the intervention, examiner 1 will return to the evaluation room and repeat the same evaluation performed before the intervention.

Participants will be informed that they will receive a spinal manipulative therapy or a simulated spinal manipulative therapy and that both techniques can produce therapeutic effects. To verify the success of blinding strategies, participants will be asked after treatment what treatment they think they received, with two response options: (1) spinal manipulation treatment or (2) sham spinal manipulation treatment.

### Intervention

The protocol will be performed by a physiotherapist blinded to the initial assessment and will be conducted according to the requirements Template for Intervention Description and Replication (TIDieR) [31].

#### Spinal manipulative therapy

Spinal manipulative therapy will be performed using the technique called *lumbar roll* by a physiotherapist with 10 years of clinical experience*.* The patient will be positioned in lateral decubitus with the target side up, knee flexed, and lower hip extended; the physiotherapist will stabilise the shoulder with the cephalic hand and the thigh with his leg and make manual contact with the caudal hand over the process nipple on the upper side of the vertebra to be manipulated with the hypothenar region of the caudal hand. The manipulation will be performed with a passive rotation movement at high speed and low amplitude in the posteroanterior direction in association with the fall of the applicator body [18, 32]. The intervention will be carried out in a single moment. The manipulation will be carried out bilaterally, starting from the symptomatic side. The treatment will be considered complete in the presence of joint noise or after two attempts with no joint noise.

#### Simulated spine manipulation

The simulation of the spinal manipulative technique will be based on current recommendations [33]. The simulated technique will be performed similarly to actual manipulation by the same physiotherapist, but with manual contact of the physiotherapist with the superior medial gluteal musculature in a wide and nonspecific way with the hand palm. The participant’s spine will be kept in a neutral position and 90° of hip flexion. The applicator will perform a slow, smooth, and unspecific impulse associated with a small fall of the body [15,18]. The simulated technique will be carried out bilaterally for two times, starting from the symptomatic side.

### Criteria for discontinuing allocated interventions

The trial will be discontinued in case of serious adverse events (any significant disability, hospitalisation, life-threatening, and death) occur that make continuing the study harmful for the participants regardless if related to the intervention (or control) or not.

### Ancillary and post-trial care

Ancillary and post-trial care (e.g. provision and/or cover for additional health care of immediate adverse events related to trial procedures) will be provided for participants who suffer sustained harm due to their involvement in this trial at no costs. All participants will receive physiotherapy treatment as usual after participating in the study.

### Outcome measures

The primary outcomes will be pressure pain threshold and postural instability. Secondary outcomes will include pain intensity and patient’s expectation.

#### Primary outcome variables

##### Evaluation of the pain threshold

The evaluation of the pressure pain threshold will be performed by a trained evaluator using a digital pressure algometer with a 1-cm^2^ rubber probe. The device will be properly calibrated. The evaluation points will be measured bilaterally three times in the following places: the middle portion of the calf in the medial gastrocnemius muscle, anterior tibial muscle laterally at the level of the anterior tibial tuberosity, 2 cm laterally to the L5 spinous process, 2 cm laterally to the spinous process of L1, the medium portion of the deltoid muscle, and 2 cm distal to the lateral epicondyle. The probe will be placed perpendicular to the skin, and the pressure increased at a rate of 500 g/s while the examiner visually monitors the force in real time by reading the digital display. The participant will be instructed to say “stop” as soon as the pressure sensation becomes painful, the applicator will remove the algometer, and the threshold will be recorded electronically. For data analysis, the average of the three values obtained in each evaluation measure by pressure algometer in pre- and post-intervention will be used [34]. The minimal important clinical difference in the assessment of the pressure pain threshold considered will be at least 15% between pre- and post-intervention. The use of an algometer to assess the pressure pain threshold is considered to have excellent reproducibility and validity [35]. A study described inter-rater reliability for pressure pain threshold, and highly reliable measures can be found when pressure pain threshold is calculated as the mean of 3 measures [35].

##### Evaluation of postural stability

The evaluation of postural stability will be performed in a baropodometry exam by displacing the CoP through the platform *FootWork*, with an active surface of 400 × 400 mm dimensions of 645×520×25 mm, and a USB-powered connection connected to a notebook. The evaluation will be carried out in a specific task with eyes open. Participants must remain standing on the platform keeping their eyes fixed on a mark 2 m away. The participant will be instructed to remain static, in an anatomical position with feet spaced at hip-width with elbows extended along the trunk, holding in each hand a bag that weighs 2 kg. During the examination, participants will remain barefoot on the baropodometer. The participant will then be instructed to perform as many as possible squats in 40 s. The stability variable investigated using the baropodometer will be the area of the CoP ellipse (A-CoP in millimetres squared). Improved postural stability is assessed by decreasing the oscillation of the CoP through baropodometry from the observation of a variation of at least 15% in the pre- and post-intervention measurements. A previous study carried out by our group showed that the barodometer is a valid instrument to measure postural stability through CoP (sway area) displacement in patients with nonspecific cLBP [36].

#### Secondary outcome variables

##### Pain intensity

Pain intensity will be measured using the Numeric Pain Rating Scale (NPRS) from 0 (no pain) to 10 (worst possible pain) before and immediately after the intervention in both groups. A clinically significant decrease in pain intensity assessed by the NPRS will be considered if a variation of at least 2 points between the pre- and post-intervention assessment is observed [37].

##### Patient expectation

We will use a Likert scale to assess the patient’s expectation regarding the treatment: the patient will be asked after the intervention about their expectation of the intervention with the following question: If you think about how you felt before the treatment, how you expected it to look after treatment: (1) worse, (2) a little worse, (3) neither better nor worse, (4) a little better, and (5) much better.

### Serious adverse events and adverse events

The assessment of serious adverse events and adverse events that occurred during the intervention will be assessed by a self-reported questionnaire, including the symptom and/or adverse event, with duration and intensity details. Serious adverse events will include any significant disability, hospitalisation, life-threatening, and death and will be reported immediately to the researchers and ethics committee.

### Data collection and management

Patient’s characteristics at baseline assessment will be collected immediately before randomisation. All the data collected, and outcomes will be obtained using printed questionnaires.

#### Baseline assessment

**Fig. 1:**
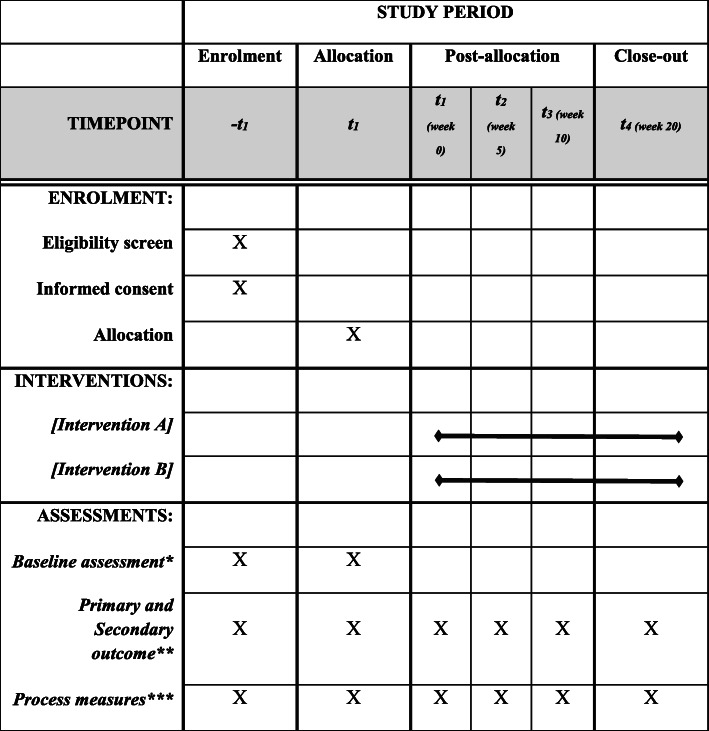
Schedule of enrolment, interventions, and assessments. *Sociodemographic, clinical, personal, and anthropometric characteristics. **Primary outcome: pressure pain threshold and postural instability. Secondary outcome: pain intensity and patient’s expectation. ***Adverse events

A self-administered questionnaire will be filled out by the participants regarding sociodemographic characteristics and personal data (age, sex, marital status, profession, education level, address, telephone, e-mail) and anthropometric data (weight, height, and body mass). Subsequently, body mass index (BMI) will be calculated by weight in kilogrammes divided by height in metres squared (kg/m^2^). BMI categories will be divided into underweight (BMI < 18.5), normal weight (BMI 18.5 to 24.9), overweight (BMI 25.0 to 29.9), and obese (BMI ≥30.0). The schedule will be performed as presented in Fig. 1.

#### Data management

The original data will be scanned as image files by a research assistant and shared with a second research assistant. All data will be stored in a password-protected computer and data integrity will be checked regularly for omissions and errors by double entered with automated checks in Microsoft Excel spreadsheet (Microsoft Corporation) performed remotely by an independent examiner. Discrepancies will be resolved by checking the original data.

#### Confidentiality

Participants will be identified by an individual trial number to ensure confidentiality, and confidentiality regarding the data collected in all stages will be guaranteed.

### Statistical analysis

#### Sample size calculation

The sample size calculation was performed a priori in the software G * Power version 3.1 (Heinrich-Heine-Universität, Düsseldorf, Germany) to determine a sufficient sample size. According to a model used previously [18] in individuals with LBP to detect a minimum difference of 15% (effect size of 0.64) in the low-pressure pain threshold, the statistical power of 80%, and an alpha of 0.05 in a two-way analysis of variance (ANOVA) repeated measures, the estimated sample size was 40 patients per group. A total of 80 participants will be included in the present study.

#### Data analysis

The results will be tabulated in Microsoft Excel software and analysed by an independent researcher. The results of the descriptive analysis will be presented in mean and standard deviation (SD) for continuous variables, and absolute values and proportions (%) for categorical variables. The analysis of the data distribution on primary outcomes will be performed using the Shapiro-Wilk test. The comparison between the groups regarding the effect of interventions on pressure pain threshold, postural stability, and pain intensity will be performed by two-way ANOVA. The Bonferroni post hoc test will be used when a significant *F* value is found. For each ANOVA, the metric of interest will be bidirectional interaction (intervention group x evaluation time). All statistical tests will be two-tailed with the pre-established significance level at *p* < 0.05. All data will be analysed using the JASP version 0.14.1 software, and graphics analysis will be performed using GraphPad Prism software (GraphPad Software, San Diego, CA, USA) version 8.00 for MacBook.

### Subgroup analyses

No subgroup analysis is planned for this study.

### Plans for communicating important protocol amendments to relevant parties

Important protocol modifications such as changes to eligibility criteria, outcomes, or analyses will be notified to relevant parties (e.g. Research Ethics Committee, researchers, participants, and journal of publication).

### Public and patient involvement

Public and patients were not involved in the study design. We will invite patients to be involved in the development of dissemination strategies.

### Dissemination plans

Data will be made available upon request to the researchers responsible for the study. The results will be disseminated through presentations at a scientific congress, as well as published in an indexed, peer-reviewed journal.

## Discussion

This study will provide insights regarding the immediate effects of spinal manipulation in patients with cLBP against a simulated spinal manipulation using objective outcomes and considering patients’ expectations regarding the treatment. We will assess pressure pain threshold and postural stability and the potential impact of pain intensity and patient expectations on the objective measures.

Assessing whether the immediate effects of spinal manipulation are beneficial to patients with cLBP and whether these benefits are superior to placebo is vital since patients with LBP consider immediate relief to be an acceptable outcome of their treatment. Transitory pain relief may favour the practice of exercise, which is an effective intervention for chronic low back pain. A strength of this clinical trial is to assess objective outcomes, as the placebo effect less influences them. Likewise, addressing the patient expectations regarding the treatment may shed light on possible mechanisms involving spinal manipulative therapy. On the other hand, an absence of a follow-up period represents a shortcoming of the current study. Nonetheless, it is unclear whether spinal manipulation is significantly superior over placebo on the objective outcomes acutely. Accordingly, adding follow-up periods would be time-consuming, add extra costs incurred by the patients, and require appropriate human and organisational structure resources with no clear assertion.

## Trial status

The current protocol is version 4. Recruitment started in November 2021, and it is expected to end in June 2022.

## Data Availability

Data will be made available from the principal author upon reasonable request.

## Abbreviations

ANOVA: Analysis of variance
BMI: Body mass index
cLBP: Chronic low back pain
CONSORT: Consolidated Standards of Reporting Trials
CoP: Centre of pressure
LBP: Low back pain
NPRS: Numeric Pain Rating Scale
REBEC: Brazilian Registry of Clinical Trials
SD: Standard deviation
SPIRIT: Standard Protocol Items: Recommendations for Interventional Trials
TIDieR: Template for Intervention Description and Replication
UNICENTRO: Midwestern Parana State University
UNIGUAIRACA: Guairacá University Centre
